# Vitamin D supplementation: still a challenge for rheumatologists!

**DOI:** 10.1016/j.ero.2025.02.001

**Published:** 2025-03-24

**Authors:** Willem F. Lems, Johannes W.J. Bijlsma

**Affiliations:** 1 Department of Rheumatology, Amsterdam UMC, Amsterdam, The Netherlands; 2 Department of Rheumatology, UMC Utrecht, Utrecht, The Netherlands

## Abstract

There is consensus around vitamin D in patients with osteoporosis: considerable data have shown that vitamin D may be favourable in the elderly because of a positive effect on bone health and muscle strength. However, in the field of rheumatology, there are still unresolved items: (i) low vitamin D levels are associated with higher disease activity in patients with inflammatory arthritis (rheumatoid arthritis [RA], systemic lupus erythematosus [SLE], spondyloarthritis [SpA], and psoriatic arthritis [PsA]) and (ii) vitamin D supplementation (2000 IU/d) seems to reduce the risk of new inflammatory diseases. We discussed these data because they are new and relevant for rheumatologists in their daily practice, and the potential favourable effects of higher dosages of vitamin D in patients with inflammatory arthritis should be high on the research agenda.

## INTRODUCTION

In recent years, the value of vitamin D supplementation to prevent fractures has been challenged: 2 randomised controlled trials (RCTs) have been published, both with more than 20,000 participants, that failed to show any effect of high-dose vitamin D supplementation (2000 IU/d) on fracture reduction in elderly ambulatory individuals with adequate vitamin D levels [[1], [2], [3]]. This may lead to the oversimplified conclusion that vitamin D supplementation is not necessary. We will argue that many patients with inflammatory rheumatic diseases (IRDs) might still benefit from vitamin D supplementation and suggest that the role of (adjuvant) vitamin D (a very cheap and safe drug) in disease activity and occurrence of IRDs is still worthwhile to explore further.

## VITAMIN D AND FRACTURES AND FALLS IN ELDERLY

Adequate vitamin D levels are crucial for the elderly. Low levels of 25-hydroxy (25-OH) vitamin D often occur in the elderly, and vitamin D deficiency is associated with osteoporosis and fractures as well as with muscular weakness and frailty. For over 30 years, we have known that vitamin D supplementation in the elderly has favourable effects on hip-fracture reduction: in ambulatory elderly patients living in a nursing home with a mean age of 84 years, hip fractures were reduced by 43% [4]. In 2005, a meta-analysis of 5 RCTs in elderly patients indicated that vitamin D supplementation with 700 to 800 IU/d reduces both hip and nonvertebral fractures by 26% and 23%, respectively. This was not shown with supplementation of 400 IU/d [5]. In addition, it was shown that supplemental vitamin D in a dose of 700 to 1000 IU/d also reduces falls by approximately 20% [6].

## 25-OH VITAMIN D LEVELS AND DISEASE ACTIVITY IN PATIENTS WITH IRDs

Patients with IRDs are at risk of vitamin D deficiency, probably due to immobility and lack of exposure to sunshine (such as in systemic lupus erythematosus [SLE]). 25-OH vitamin D levels were found to be lower in patients with rheumatoid arthritis (RA) than in healthy controls (52.7 nmol/L vs 82.3 nmol/L), and remarkably, an inverse relationship between 25-OH vitamin D levels and disease activity was observed: 88 nmol/L in patients in remission, 56.2 nmol/L in patients with moderate disease activity, and (only) 31.5 nmol/L in patients with high disease activity [7]. In a meta-analysis of 24 studies including 2148 patients with RA and 1991 healthy controls, patients with RA had lower 25-OH vitamin D levels than healthy controls (difference, 16.5 nmol/L), and the negative relationship between 25-OH vitamin D levels and disease activity of RA was confirmed [8].

Similar results were found in patients with SLE: mean serum level of 25-OH vitamin D was 51.5 nmol/L, more than 80% had insufficient 25-OH vitamin D levels (here defined as <75 nmol/L), and low 25-OH vitamin D levels were associated with high disease activity [9]. In a meta-analysis comparing 503 patients with spondyloarthritis (SpA) and 398 healthy controls, 25-OH vitamin D levels were lower (18.8 nmol/L) in the patients with SpA and a negative correlation was found between 25-OH vitamin D levels and erythrocyte sedimentation rate (ESR), C-reactive protein (CRP), and Bath Ankylosing Spondylitis Disease Activity Index (BASDAI) [10]. A comparable difference (16 nmol/L) was found in a recent systematic review of 264 patients with psoriatic arthritis (PsA) vs 287 healthy controls [11].

Rheumatologists should be alert on vitamin D deficiency in users of glucocorticoids (GCs): the risk of low 25-OH vitamin D of <25 nmol/L was doubled in GC users compared with nonusers [12]. It is not fully elucidated whether this is related to lack of exposure to sunshine or to metabolic changes in vitamin D metabolites induced by GCs. European Alliance of Associations for Rheumatology (EULAR) recommendations on responsible use of GCs supported the use of vitamin D in combination with calcium [13,14].

## VITAMIN D AND INFLAMMATION IN PATIENTS WITH IRD

Although reliable data exist on the prevention of osteoporotic fractures with vitamin D, the role of vitamin D in inflammation in patients with IRD is far from elucidated. The discovery of vitamin D receptor in inflammatory cells and observations that several inflammatory cells produce vitamin D suggest that vitamin D has immunomodulating properties [15]. In a recent landmark article, Cutolo et al [16] showed that 1,25-OH vitamin D_3_, the active form of vitamin D, can modulate the innate immune system in response to invading pathogens, downregulate inflammatory responses, and support the adaptive arm of the immune reaction.

Low levels of 25-OH vitamin D in patients with IRDs and an inverse relationship between disease activity and vitamin D levels have been mentioned above. An interesting finding was reported in a 5 years study (ref [1]) with ambulatory etc. (1): in this 5-year study with ambulatory elderly patients receiving 2000 IU of vitamin D or placebo daily, no difference was found in the occurrence of incident autoimmune diseases in the first 3 years, but in years 4 and 5, the lines diverged: overall, there was a 22% reduction in incident autoimmune diseases, mostly RA and polymyalgia rheumatica (PMR) (95% CI, 0.61-0.99), in the vitamin D arm [17]. During the follow-up in years 6 and 7, when vitamin D and placebo were stopped, the lines completely converged again [18].

Although several studies have recently been performed with GCs, methotrexate (MTX), rituximab, and abatacept in order to prevent the occurrence of clinical RA in patients with arthralgia and risk factors for RA, all drugs with considerable side effects, these data from the VITAL study support the suggestion that vitamin D might be an attractive (and safe) option for research for this indication.

Clinical data on the effects of vitamin D supplementation in patients with IRDs are scarce: in a systematic review of 11 studies, 360 patients with RA were treated with varying dosages of vitamin D: ESR, CRP, and Disease Activity Score (DAS)-28 joint score were not influenced by treatment with vitamin D, but visual analogue scale (VAS) pain score, DAS-CRP, and DAS-ESR were reduced in the vitamin D group. In addition, higher dosages had a stronger effect on lowering CRP than lower dosages [19]. These data should be interpreted with caution because there are differences in vitamin D dosage and vitamin D baseline levels and because half of the studies were open-label [19].

## SUMMARY

Vitamin D dosages (800–1000 IU/d) prevent falls and fractures in patients at risk of osteoporotic fractures [20], in those with a low 25-OH vitamin D level, which should proposal to above 50 nmol/L [[21], [22], [23]] or eventually up to 75 nmol/L [24]. For rheumatologists, there are challenges in daily practice: patients with prevalent IRDs have an elevated risk of low vitamin D levels, and low vitamin D levels are associated with an increase in falls and fractures and possibly with higher disease activity in patients with IRDs.

Diagnosing suboptimal vitamin D levels and supplementation with dosages that are adequate for fracture prevention, 800 to 1000 IU/d, is therefore the first step. It is a research question whether higher dosages of adjuvant vitamin D have an anti-inflammatory effect in patients with IRDs, and even more challenging is the question whether we can prevent or postpone the occurrence of IRDs with vitamin D.

## CRediT authorship contribution statement

**Willem F. Lems:** Writing – original draft, Conceptualization. **Johannes W.J. Bijlsma:** Writing – original draft, Conceptualization.
